# Cocaine Effects on Dopaminergic Transmission Depend on a Balance between Sigma-1 and Sigma-2 Receptor Expression

**DOI:** 10.3389/fnmol.2018.00017

**Published:** 2018-02-12

**Authors:** David Aguinaga, Mireia Medrano, Ignacio Vega-Quiroga, Katia Gysling, Enric I. Canela, Gemma Navarro, Rafael Franco

**Affiliations:** ^1^Centro de Investigación en Red, Enfermedades Neurodegenerativas (CIBERNED), Instituto de Salud Carlos III, Madrid, Spain; ^2^Department of Biochemistry and Molecular Biomedicine, School of Biology, Universitat de Barcelona, Barcelona, Spain; ^3^Department of Cellular and Molecular Biology, Faculty of Biological Sciences, Pontificia Universidad Católica de Chile, Santiago, Chile; ^4^Department of Biochemistry and Physiology, Faculty of Pharmacy, Universitat de Barcelona, Barcelona, Spain

**Keywords:** acute, addiction, cAMP, chronic, dopamine D_1_ and D_2_ receptors, ERK1/2 phosphorylation, label-free, signaling

## Abstract

Sigma σ_1_ and σ_2_ receptors are targets of cocaine. Despite sharing a similar name, the two receptors are structurally unrelated and their physiological role is unknown. Cocaine increases the level of dopamine, a key neurotransmitter in CNS motor control and reward areas. While the drug also affects dopaminergic signaling by allosteric modulations exerted by σ_1_R interacting with dopamine D_1_ and D_2_ receptors, the potential regulation of dopaminergic transmission by σ_2_R is also unknown. We here demonstrate that σ_2_R may form heteroreceptor complexes with D_1_ but not with D_2_ receptors. Remarkably σ_1_, σ_2_, and D_1_ receptors may form heterotrimers with particular signaling properties. Determination of cAMP levels, MAP kinase activation and label-free assays demonstrate allosteric interactions within the trimer. Importantly, the presence of σ_2_R induces bias in signal transduction as σ_2_R ligands increase cAMP signaling whereas reduce MAP kinase activation. These effects, which are opposite to those exerted via σ_1_R, suggest that the D_1_ receptor-mediated signaling depends on the degree of trimer formation and the differential balance of sigma receptor and heteroreceptor expression in acute versus chronic cocaine consumption. Although the physiological role is unknown, the heteroreceptor complex formed by σ_1_, σ_2_, and D_1_ receptors arise as relevant to convey the cocaine actions on motor control and reward circuits and as a key factor in acquisition of the addictive habit.

## Introduction

In advanced societies cocaine addiction is an important health and socio-economic problem. Cocaine use begins recreationally and the seeking behavior is based on a feeling of general well-being. Drug addiction is the result of plastic changes in areas of the brain that have dopamine as the main neurotransmitter, particularly in the *ventral tegmental area* (VTA) (see Lüscher, 2013 and references therein). The main consequence of cocaine consumption in the central nervous system (CNS) is an increase in interneuronal dopamine levels, which is not limited to VTA but extends to other structures, such as the basal ganglia (Wise, 1984; Bradberry, 2008). It was thought that the inhibition of dopamine transporters was at the root of all the effects caused by this drug of abuse. However, there is strong evidence showing that cocaine exerts effects by a direct interaction with sigma receptors. Two different sigma receptors have been identified that are functionally and structurally unrelated. Although endogenous ligands are not known and the physiological function of sigma receptors is unclear, these receptors share the ability to bind cocaine. On the one hand, sigma-1 receptor (σ_1_R) is a chaperone that spans once the membrane bilayer and whose recently reported structure consists of a homotrimer (Schmidt et al., 2016). On the other hand, sigma-2 receptor (σ_2_R) was identified as a member of the family of membrane-associated progesterone receptors; apart from σ_2_R (PGRMC1), three other human members are identified: PGRMC2, neuferricin, and neudesin. They are haem proteins displaying a cytochrome b_5_-fold domain. While σ_2_R dimerization affects proliferation and chemoresistance in tumor/metastasis *in vitro* models and xenograft- based tumor/metastasis models, the mode of action in the periphery and the CNS are virtually unknown (Kabe et al., 2016; reviewed in Cahill, 2017). The interaction of σ_1_R with dopamine receptors and the relevant role that σ_1_R exerts on the modulation of dopaminergic signaling by cocaine has been reported. In contrast, no study has been undertaken to know whether the binding of cocaine to σ_2_R results in dopaminergic regulation.

The role of σ_1_R as relevant target of cocaine was suspected due to the moderate affinity of drug binding to the receptor (Matsumoto et al., 2003; Hayashi and Su, 2005). Therefore, it seems that the “physiologically” relevant concentrations of cocaine can both inhibit the uptake of dopamine and activate σ_1_R. σ_1_R-cocaine interaction intervenes in the triggering of locomotor and convulsive actions of the drug (Menkel et al., 1991; Matsumoto et al., 2001a,b, 2002; Barr et al., 2015). In addition, synthetic drugs that act as σ_1_R agonists and antagonists, respectively, potentiate (Matsumoto et al., 2002, 2003) and reduce (Matsumoto et al., 2004) cocaine actions. More recent studies have identified in both heterologous expression systems and natural sources an interaction between σ_1_R and dopamine receptors (Navarro et al., 2010; Moreno et al., 2014; Borroto-Escuela et al., 2017). Accordingly, it has been suggested that dual antagonism of σ_1_R and inhibition of the dopamine DAT transporter can effectively block cocaine self-administration (Katz et al., 2016). Matsumoto et al. (2007) reported that treatment with synthetic drugs that act on σ_2_R attenuates cocaine-derived behavior in mice. Although the selectivity of the compounds was poor, 1 year later, Mésangeau et al. (2008) designed an approach for converting selective σ_1_R ligands into σ_2_R selective ligands that, importantly, showed anti-cocaine activity. Furthermore, it has been observed that treatment with σ_2_R antagonists counteract locomotor stimulation induced by cocaine in mice (Lever et al., 2014; Guo and Zhen, 2015).

An important physiological consequence of cocaine ingestion is an increase in motor activity, which is controlled by basal-ganglia brain circuits. Motor control is exerted by the direct and indirect pathways of the basal ganglia and associated nuclei. Of the five types of dopamine receptors, the D_1_ (D_1_R) is enriched in the direct pathway, while the D_2_ (D_2_R) is enriched in the indirect route. The balance of the dopaminergic input in the two circuits results in fine-tuning motor control. The locomotor hyperactivity resulting from cocaine use probably reflects a lack of balance in these two routes. The objective of this work was to investigate how the binding of cocaine to σ_2_R affects dopaminergic signaling mediated by D_1_R and/or D_2_R. We first investigated whether σ_2_R interacts with D_1_R or with D_2_R and, subsequently, we observed how cocaine could affect in a σ_1_R-independent but σ_2_R-dependent fashion the signal transduction triggered by agonist activation of D_1_R but not of D_2_R.

## Results

### σ_2_R May Form Complexes with Dopamine D_1_ But Not with Dopamine D_2_ Receptors

**FIGURE 1:**
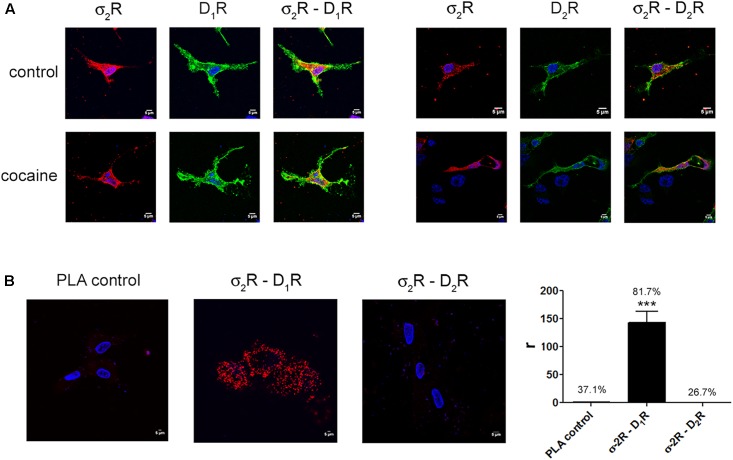
Expression of σ_2_R-containing heteromer complexes in a heterologous expression system. To determine colocalization between σ_2_R and dopamine D_1_ or D_2_ receptors, immunocytochemistry assays were performed in HEK-293T pretreated or not with 30 μM cocaine for 30 min. HEK-293T cells expressing σ_2_R-Rluc (1 μg cDNA), D_1_R-YFP (1 μg cDNA), D_2_R-YFP (1 μg cDNA), σ_2_R-Rluc (1 μg cDNA) and D_1_R-YFP (1 μg cDNA) or σ_2_R-Rluc (1 μg cDNA) and D_2_R-YFP (1 μg cDNA) were used. Dopamine receptors were detected by YFP fluorescence (green) and σ_2_R was detected by a specific antibody against Rluc (1/100, Millipore, Temecula, CA, United States) followed by a Cy3-conjugated secondary antibody (1/200, Jackson Immunoresearch Laboratories, West Grove, PA, United States) (red). Colocalization is shown in yellow **(A)**. Scale bar 5 μm. *In situ* proximity ligation assay (PLA) was developed in HEK-293T cells expressing D_1_R (1 μg cDNA) or D_2_R (1 μg cDNA) and σ_2_R (1 μg cDNA) by the use of specific primary antibodies (1/100 dilution) against D_1_R, D_2_R and/or σ_2_R. Nuclei were stained with Hoechst (1/100). Confocal microscopy images (four superimposed sections) were obtained showing D_1_R-σ_2_R or D_2_R-σ_2_R complexes as red spots **(B)**. Scale bar 5 μm. Quantification of the PLA provides in the Y-axis the ratio *r* (number of red spots/cell containing spots) and, above each bar, the percentage of positive cells versus the total number of cells (blue nucleus). Data are the mean ± SEM of four different fields in five independent preparations. One way ANOVA and Dunnett’s *post hoc* test showed statistically significant differences (^∗∗∗^*p* < 0.001).

**FIGURE 2:**
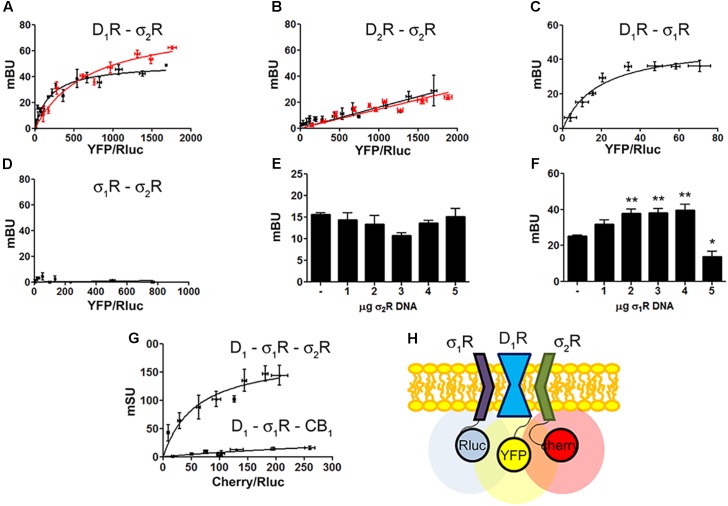
Identification of D_1_R-σ_1_R-σ_2_R heteromers in a heterologous expression system. Bioluminescence energy transfer (BRET) was developed in HEK-293T cells expressing (i) a constant amount of D_1_R-Rluc (0.3 μg cDNA) **(A)** or D_2_R-Rluc (0.25 μg cDNA) **(B)** and increasing amounts of σ_2_R-YFP (0.5–4 μg cDNA), or (ii) a constant amount of D_1_R-Rluc (0.3 μg cDNA) and increasing amounts (0.5–4.5 μg cDNA) of σ_1_R-YFP **(C)** or (iii) a constant amount of σ_1_R-Rluc (0.1 μg cDNA) and increasing amounts of σ_2_R-YFP (1–6 μg cDNA) **(D)**. Cells were treated (red line) or not (black line) with 30 μM cocaine for 30 min. BRET is expressed as milli BRET units (mBU) and is given as the mean ± SEM of seven different experiments. Competition experiments were developed in HEK-293T cells expressing a constant amount of σ_1_R-Rluc (0.1 μg cDNA) and D_1_-YFP (1.5 μg cDNA) and increasing amounts of unfused σ_2_R (0–5 μg cDNA) **(E)** or a constant amount of D_1_-Rluc (0.05 μg cDNA) and σ_2_R-YFP (0.3 μg cDNA) and increasing amounts of unfused σ_1_R (0–5 μg cDNA) **(F)**. Transfer of energy was expressed as milli BRET units (mBU) and results are given as the mean ± SEM of 10 different experiments. One way ANOVA and Dunnett’s *post hoc* test showed statistically significant differences (^∗^*p* < 0.05, ^∗∗^*p* < 0.01). **(G)** Sequential resonance energy transfer (SRET) assay developed in HEK-293T cells transfected with constant amounts of σ_1_R-Rluc (0.2 μg cDNA) and D_1_R-YFP (1.5 μg cDNA) and increasing amounts of σ_2_R-RFP (0.5–4 μg cDNA). A negative control was performed using cDNA for the cannabinoid CB_1_ receptor (fused to RFP) instead of cDNA for σ_2_R-RFP. SRET is expressed as milli SRET units (mSU) and are given as the mean ± SEM of 6 different experiments. **(H)** Schematic representation of SRET.

Two different sigma receptors have been described, the non-opioid receptor, σ_1_R, and the PGRMC-1 protein, also known as σ_2_. Despite the endogenous ligands are not known, the two sigma receptors may bind cocaine. While recent studies have demonstrated that σ_1_R is involved in cocaine modulation of dopamine receptor function, a similar study on σ_2_R-mediated modulation of dopaminergic signaling is lacking. We first evaluated in a heterologous expression system whether σ_2_R may colocalize with dopamine receptors at the plasma membrane. Immunocytochemistry assays were undertaken in HEK-293T cells expressing σ_2_R fused to Rluc and either dopamine D_1_R fused to YFP or dopamine D_2_R fused to YFP. The σ_2_R expression was identified by a specific antibody against Rluc protein and a secondary Cy3 antibody, while dopamine receptor-YFP expression was identified by its own fluorescence. D_1_R (green) was detectable at the plasma membrane level while σ_2_R (red) was expressed both in intracellular structures and at the plasma membrane, where it colocalized (yellow) with D_1_R (**Figure 1A**, left images). When a similar experiment was developed with D_2_R, similar results were obtained indicating that D_2_R and σ_2_R colocalize at the cell surface (**Figure 1A**, right images). When the immunocytochemical assays were performed in cells pretreated with 30 μM cocaine for 30 min, the level of colocalization between σ_2_R and D_1_R or D_2_R was similar, indicating that cocaine pretreatment did affect neither cell surface expression of σ_2_R, D_1_R or D_2_R nor receptor colocalization. Next, we determined whether σ_2_R may form heteromer complexes with dopamine D_1_ or D_2_ receptors. For this purpose, we took advantage of energy transfer assays and *in situ* proximity ligation assay (PLA), which allows the identification of close proximity between two proteins (<17 nm) (Borroto-Escuela et al., 2011; Trifilieff et al., 2011). For PLA, HEK-293T cells expressing σ_2_R and either D_1_R or D_2_R were treated with specific primary antibodies against σ_2_R and against each of the dopamine receptors. Interestingly, the red punctuated signal around Hoechst-stained nuclei was much higher for D_1_R and σ_2_R than for D_2_R and σ_2_R (82 versus 27% of labeled cells) (**Figure 1B**). Finally, we developed bioluminescence energy transfer assays in HEK-293T cells transfected with cDNAs for D_1_R-Rluc or D_2_R-Rluc and increasing amounts of cDNA for σ_2_R-YFP. Interestingly, a saturable BRET curve was obtained (BRET_max_ 50 ± 3, BRET_50_ 190 ± 40) (**Figure 2A**) indicating a specific interaction between D_1_R-σ_2_R; in contrast, a linear signal was obtained between D_2_R-σ_2_R (**Figure 2B**) suggesting a lack of interaction between them. When the same experiments were undertaken in cells treated with cocaine, similar results were obtained for the D_1_R-Rluc/σ_2_R-YFP donor/acceptor pair (BRET_max_ 82 ± 10, BRET_50_ 680 ± 200), indicating that cocaine did not significantly affect the interaction (**Figure 2B**).

### Dopamine D_1_R, σ_1_R, and σ_2_R May Form Heterotrimeric Complexes

Dopamine D_1_ and σ_1_ receptors may form heteromeric complexes in HEK-293T cells (Navarro et al., 2010). To confirm whether in our experimental conditions D_1_R-Rluc may act as a donor of σ_1_R-YFP, BRET experiments undertaken in cotransfected HEK-293T cells provided a saturable curve thus indicating the interaction between σ_1_R and D_1_R (**Figure 2C**). We then hypothesized that σ_1_R and σ_2_R could be interacting together. Accordingly, BRET assays were performed in HEK-293T cells expressing a constant amount of σ_1_R-Rluc and increasing amounts of σ_2_R-YFP. The unspecific linear signal obtained (**Figure 2D**) suggested that no interaction was occurring between the two sigma receptors. We then performed assays to investigate whether σ_1_R and σ_2_R competed for the binding to D_1_R. BRET experiments were then developed in HEK-293T cells expressing a constant amount of σ_1_R-Rluc and D_1_-YFP and increasing amounts of non-fused σ_2_R. The results indicated that σ_2_R was not able to compete with σ_1_R for heteromer formation since the energy transfer between donor and acceptor was not altered (**Figure 2E**). When a similar experiment was performed expressing a constant amount of D_1_-Rluc and of σ_2_R-YFP and increasing amounts of non-fused σ_1_R, the results indicated that low expression levels of σ_1_R increased BRET signals; however, higher expression levels σ_1_R were able to displace σ_2_R out of the heteromer, as reflected by a significant decrease in BRET signal (**Figure 2F**). This result could reflect the formation of D_1_R-σ_1_R-σ_2_R heterotrimer complexes, where the interaction of σ_1_R to the σ_2_R-D_1_R complex could create a structural change in turn leading to increasing the energy transfer between Rluc and YFP. To confirm this possibility, sequential resonance energy transfer (SRET) assays, which permits detection of trimers (Carriba et al., 2008), were developed in HEK-293T cells expressing a constant amount of σ_1_R-Rluc and of D_1_R-YFP and increasing amounts of σ_2_R-Cherry. The saturable SRET curve indicates that formation of σ_1_R-D_1_R-σ_2_R heteromer complexes was occurring (**Figures 2G,H**). The negative control was performed by substituting σ_2_R-RFP by the cannabinoid CB_1_ receptor fused to RFP thus confirming the specificity of the triple σ_1_R-D_1_R-σ_2_R interaction (**Figure 2G**).

### σ_2_R Activation Blocks Dopamine D_1_R Signaling

**FIGURE 3:**
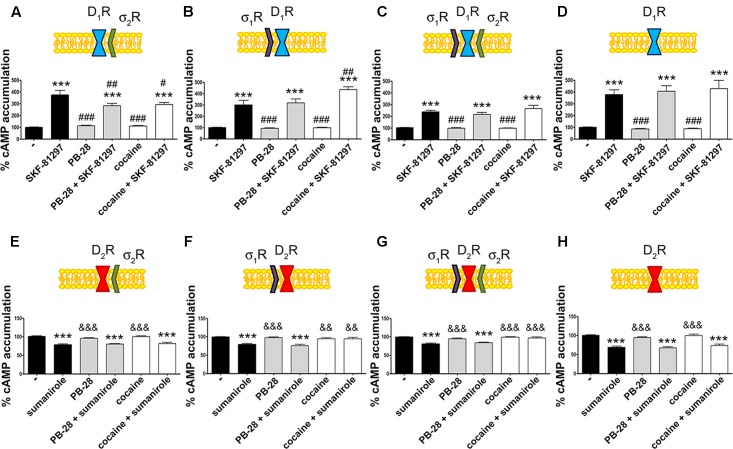
σ_2_R modulation of D_1_ receptor-mediated signaling in a heterologous expression system. cAMP determination experiments were developed in HEK-293T cells expressing D_1_R **(A–D)** or D_2_R **(E–H)** in the absence **(C,G)** or presence of 3 μg siRNA for σ_1_R **(A,E)**, 3 μg siRNA for σ_2_R **(B,F)** or both **(D,H)**. Cells were pretreated with 30 μM cocaine for 30 min, 300 nM PB-28 or vehicle 15 min prior to receptor activation using 200 nM SKF-81297 or 500 nM sumanirole. In cells expressing D_2_R 0.5 μM forskolin was used to induce increases in cAMP levels. Basal [cAMP] is considered 100% in cells expressing D_1_R, whereas forskolin-induced [cAMP] is considered 100% in cells expressing D_2_R. Values are the mean ± SEM of 12–15 different experiments. One way ANOVA followed by a Dunnett’s multiple comparison *post hoc* test showed a significant effect of treatments versus control (^∗∗∗^*p* < 0.001), a significant effect of treatments versus SKF-81297 (^#^*p* < 0.05, ^##^*p* < 0.01, and ^###^*p* < 0.001) and a significant effect of treatments versus sumanirole (^&&^*p* < 0.01 and ^&&&^*p* < 0.001).

Our next aim was to characterize the functionality of the σ_1_R-D_1_R-σ_2_R heterotrimer structure in HEK-293T cells treated with cocaine. It should be noted that σ_1_R (Navarro et al., 2010) and σ_2_R (Johannessen et al., 2011) are endogenously expressed in HEK-293T cells; consequently, we used a siRNA approach to silence σ_1_R or σ_2_R expression thus impeding heterotrimer formation. When HEK-293T cells were transfected with D_1_R and siRNA for σ_1_R, SKF-81297-induced a significant increase in cAMP levels, that was inhibited by pretreatment with cocaine or with the σ_2_R agonist, PB-28, indicating that cocaine decreases D_1_R-mediated cAMP signaling function through its binding to σ_2_R (**Figure 3A**). When HEK-293T cells were transfected with D_1_R and siRNA for σ_2_R, the results indicated that cocaine pretreatment potentiated agonist-induced cAMP levels, which was evidence of cocaine action upon binding to the σ_1_R (**Figure 3B**). The next set of results is consistent with a reciprocal modulation of signaling mediated by cocaine binding to σ_2_R and σ_1_R; while cocaine via σ_1_R positively modulates cAMP levels, it inhibits cAMP signaling via σ_2_R. Accordingly, no effect of cocaine was observed in HEK-293T expressing D_1_R and the two endogenous sigma receptors (**Figure 3C**). The lack of modulation exerted by cocaine upon simultaneous binding to both σ_1_R and σ_2_R likely reflects a balance which would, in a physiological set-up, depend on the relative expression of the two sigma receptors. In fact, when HEK-293T cells were transfected with D_1_R and both siRNA for σ_1_R and σ_2_R, cocaine or the specific σ_2_R agonist, PB-28, had no effect, indicating that cocaine modulation over D_1_R depends on σ_1_R and σ_2_R expression (**Figure 3D**).

We next investigated whether cocaine binding to σ_2_R receptors could still modulate D_2_R-mediated signaling. HEK-293T cells transfected with cDNAs for D_2_R and siRNA for σ_1_R, responded to the selective-D_2_R agonist, sumanirole. In these cells the G_i_-mediated decrease of forskolin-induced cAMP accumulation due to G_i_ coupling was not affected by cocaine pretreatment (**Figure 3E**). These results agree with the lack of interaction between σ_2_R and dopamine D_2_R (see **Figure 2B**). As a control, we confirmed that when the σ_1_R-cocaine modulation over D_2_R was assayed, i.e., silencing σ_2_R expression, cocaine was able to block the sumanirole-induced effect (**Figure 3F**). These results agree with those in Navarro et al. (2013) in the sense that they reflect the consequence of a physical interaction between σ_1_R and D_2_R receptors. In agreement with this hypothesis, HEK-293T cells expressing D_2_R and endogenous sigma receptors behaved as cells in which the σ_2_R was silenced (**Figure 3G**). As a further control, HEK-293T cells treated with siRNAs to silence both sigma receptors showed no modulation by cocaine over D_2_R-mediated signaling (**Figure 3H**), thus reinforcing the idea that cocaine effect over D_2_R depends on σ_1_R expression.

### σ_2_R Activation Potentiates Dopamine D_1_R MAP Kinase Phosphorylation

**FIGURE 4:**
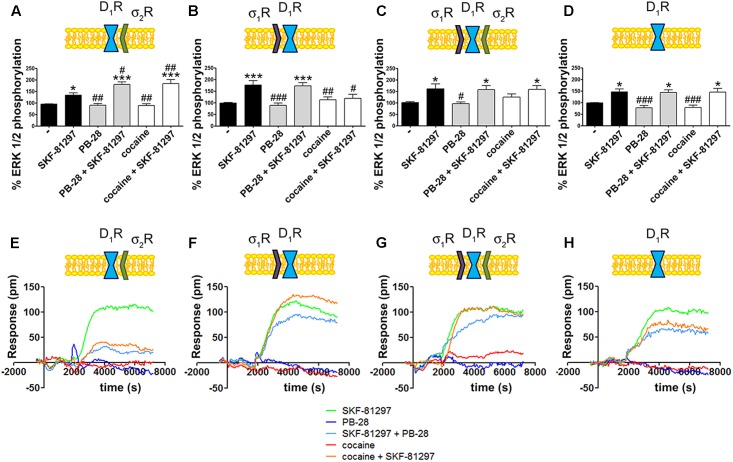
Cocaine effects on D_1_R-mediated signaling. MAP kinase activation was determined in HEK-293T cells transfected with 0.75 μg cDNA for D_1_R in the absence **(C)** or presence of 3 μg siRNA for σ_1_R **(A)**, 3 μg siRNA for σ_2_R **(B)** or both **(D)**. The culture medium was replaced by non-supplemented DMEM and 2 h later cells were treated for 30 min with 30 μM cocaine, 300 nM PB-28 or vehicle followed by a 200 nM SKF-81297 stimulation (7 min). The basal level of pERK1/2 is considered 100%. Values are the mean ± SEM of 10–12 different experiments. One way ANOVA followed by a Dunnett’s multiple comparison *post hoc* test showed a significant effect of treatments versus control (^∗^*p* < 0.05, ^∗∗∗^*p* < 0.01) and a significant effect of treatments versus SKF-81297 (^#^*p* < 0.05, ^##^*p* < 0.01, and ^###^*p* < 0.001). Real-time DMR signal 60 min recordings in HEK-293T cells transfected with 0.75 μg cDNA for D_1_R in the absence **(G)** or presence of 3 μg siRNA for σ_1_R **(E)**, 3 μg siRNA for σ_2_R **(F)** or both **(H)** that were treated with 30 μM cocaine (red), 300 nM PB-28 (dark blue) or vehicle (green) for 30 min previous to 200 nM SKF-81297 stimulation.

To further understand the cocaine effect over D_1_R function, MAP kinase signaling was evaluated in HEK-293T cells transfected with cDNAs for D_1_R and siRNA for either σ_1_R or σ_2_R. In cells expressing D_1_R with silenced σ_1_R, i.e., expressing D_1_R and σ_2_R (**Figure 4A**), cocaine pretreatment increased agonist (SFK-81297)-induced ERK1/2 phosphorylation, while in cells with silenced σ_2_R, i.e., expressing D_1_R and σ_1_R (**Figure 4B**), cocaine decreased agonist-induced ERK1/2 phosphorylation. These results are evidence of potentiation by cocaine-σ_2_R of MAP kinase signaling, and potentiation by cocaine-σ_1_R of G_s_-protein dependent signaling. In cells expressing D_1_R and the two sigma receptors, no effect of cocaine pretreatment on pERK1/2 levels was observed, in agreement with the above-described balance resulting from reciprocal sigma-receptor-mediated cocaine effects (**Figure 4C**). As a further control, cocaine did not alter the SKF-81297-induced ERK1/2 phosphorylation in HEK-293T cells expressing D_1_R and with silenced sigma receptors (**Figure 4D**). A similar experimental design was used to undertake dynamic mass redistribution (DMR) assays. DMR is a label-free technique useful to investigate the activation of G-protein coupled receptors (Grundmann and Kostenis, 2015; Medrano et al., 2017). On the one hand, in cells expressing D_1_R and σ_2_R, cocaine blocked SKF-81297-induced increase in the DMR signal in a similar way as the selective σ_2_R ligand, PB-28, did (**Figure 4E**). On the other hand, the SKF-81297 effect was potentiated by cocaine pretreatment in cells expressing D_1_R and σ_1_R (**Figure 4F**). Once more, cocaine modulation on D_1_R-agonist-induced effects was not found in cells expressing D_1_R and both sigma receptors (**Figure 4G**). As DMR in cells expressing D_1_R mainly reflects G_s_-coupling (Kebig et al., 2009; Schröder et al., 2009; Hamamoto et al., 2015), these results are similar to those obtained in cAMP read-outs. Another control was performed to show that pretreatment with the σ_2_R selective agonist, PB-28, did not result in any signal modulation in cells expressing D_1_R but silenced σ_1_R and σ_2_R expression (**Figure 4H**).

### σ_2_R Activation Blocks Dopamine D_1_R-Mediated Signaling in Primary Cultures of Striatal Neurons

**FIGURE 5:**
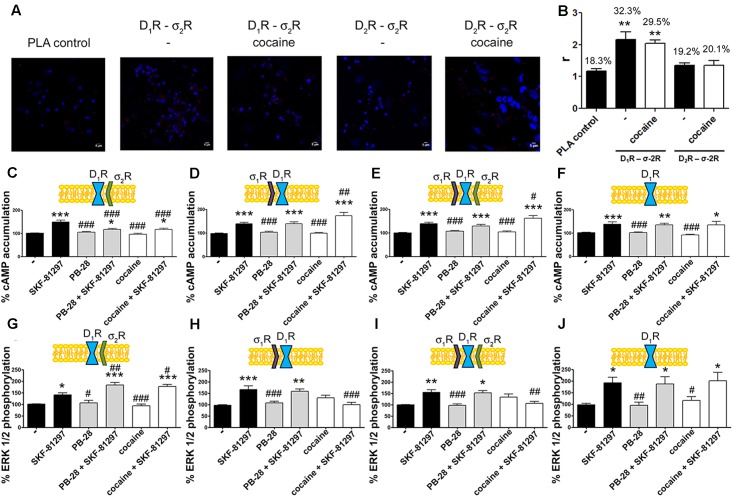
Expression and function of σ_2_R-D_1_R complexes in primary cultures of striatal neurons. In **(A,B)** PLA assay was developed in striatal primary cultures of neurons pretreated or not with cocaine 30 μM for 30 min. σ_2_R-D_1_R or σ_2_R-D_2_R heteromer complexes were detected by the use of specific antibodies (1/100 dilution) against σ_2_R and D_1_R or σ_2_R and D_2_R. Confocal microscopy images (four superimposed sections) were obtained where nuclei were stained with Hoechst (1/100). Scale bar 5 μm **(A)**. Quantification of the PLA provides in the Y-axis the ratio *r* (number of red spots/cell containing spots) and, above each bar, the percentage of positive cells versus the total number of cells (blue nucleus) **(B)**. Data are the mean ± SEM of four different fields in five independent preparations. One way ANOVA and Dunnett’s multiple comparison *post hoc* test showed statistically significant differences versus control (^∗∗^*p* < 0.01). Primary cultures of striatal neurons, control **(E,I)** or transfected with siRNA for σ_1_R **(C,G)**, σ_2_R **(D,H)** or both **(F,J)** were treated with 30 μM cocaine for 30 min or 300 nM PB-28 prior to 200 nM SKF-81297 stimulation. cAMP levels **(C–F)** or MAP kinase activation l **(G–J)** were determined. Basal [cAMP] is considered 100%. The basal level of pERK1/2 is considered 100%. Values are the mean ± SEM of 10–15 different experiments. One way ANOVA followed by a Dunnett’s multiple comparison *post hoc* test showed a significant effect of treatments versus not treated cells (^∗^*p* < 0.05, ^∗∗^*p* < 0.01, ^∗∗∗^*p* < 0.001) and a significant effect of treatments versus SKF-81297 (^#^*p* < 0.05, ^##^*p* < 0.01, and ^###^*p* < 0.001).

A proximity ligation assay (PLA) was used to determine in primary cultures of striatal neurons whether D_1_R-σ_2_R complex expression was affected by cocaine pretreatment. Consequently, specific antibodies against D_1_R and σ_2_R were used in neurons treated or not with cocaine for 30 min (**Figure 5A**). 32% of cells showed punctuated staining (with 2.2 red spots/cell containing spots) surrounding Hoechst-stained nuclei (**Figure 5B**). These results indicate the occurrence of D_1_-σ_2_ heteroreceptor complexes in striatal primary cultures of neurons. A control done in the absence of primary antibodies led to 18% of labeled cells (with 1.2 red spots/cell containing spots). The percentage of positive cells after a 30-min treatment with cocaine was around 30 (with 2 red spots/cell containing spots) (**Figure 5B**). Thus, cocaine pretreatment did not significantly alter D_1_R-σ_2_R complex formation. When PLA was developed to detect D_2_R and σ_2_R complexes, the results (19% with 1.3 red spots/cell containing spots) were similar to those in the negative control (20% with 1.4 red spots/ cell containing spots), i.e., no evidence of heteroreceptor formation was obtained. Pretreatment with cocaine did not lead to the appearance of heteromer complexes formed by D_2_R and σ_2_R (**Figure 5B**). These results agree with the BRET assays that did not find sign of interaction between the D_2_R-Rluc and σ_2_R-YFP but between the D_1_R-Rluc and σ_2_R-YFP pair.

To demonstrate the effect of cocaine over D_1_R-mediated signaling in a more physiological environment, we analyzed cAMP and MAP kinase signaling pathways in primary cultures of striatal neurons. As striatal neurons express the two sigma receptors, the siRNA approach was used to silence sigma receptor expression. On the one hand, in neurons transfected with siRNA for σ_1_R, and consequently expressing D_1_R and σ_2_R, cocaine and PB-28 led to a decrease in agonist-induced cAMP levels and to an enhancement in MAP kinase signaling (**Figures 5C,G**). On the other hand, in neurons transfected with the siRNA for σ_2_R, and consequently expressing D_1_R and σ_1_R, cocaine but not PB-28 induced an increase in the cAMP signal and a decrease in the ERK1/2 phosphorylation signal (**Figures 5D,H**). Most of these results agree with those obtained in the heterologous system. However, in striatal neurons expressing D_1_R and both sigma receptors, cocaine treatment led to a net effect that showed predominance of σ_1_R- versus σ_2_R-mediated modulation (**Figures 5E,I**). These findings could be due to a higher expression of σ_1_R-D_1_R complexes versus σ_2_R-D_1_R but they may also result from the lower affinity of the cocaine/σ_2_R binding (Lever et al., 2016). Finally, another control was performed to show that pretreatment with the σ_2_R selective agonist, PB-28, or with cocaine, did not result in any signal modulation in cells expressing D_1_R but silenced σ_1_R and σ_2_R expression (**Figures 5F,J**).

### D_1_R-Mediated Signaling Is Modulated by σ_1_R in Acute and by σ_2_R in Chronic Conditions

**FIGURE 6:**
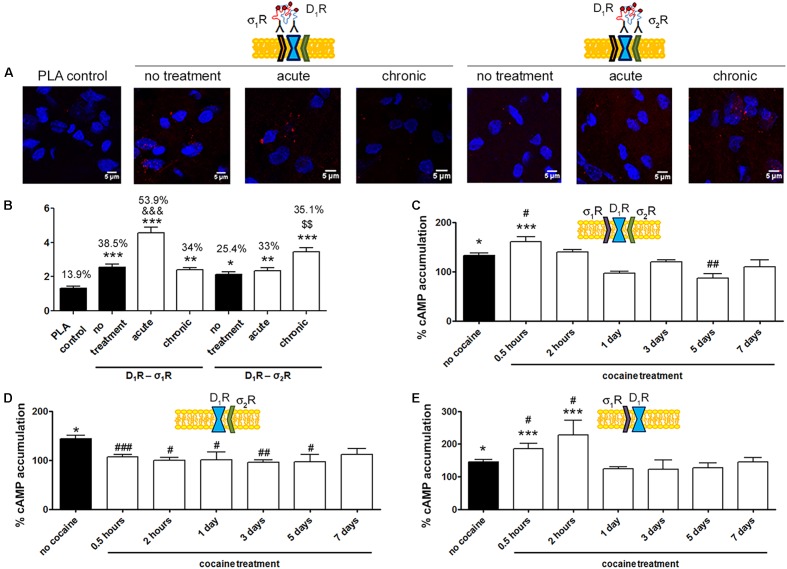
D_1_R-mediated signaling is modulated by σ_1_R in cocaine acute and by σ_2_R in cocaine chronic exposure. In **(A,B)** PLA assay was developed in brain sections from male Sprague–Dawley rats i.p. injected with vehicle or 15 mg/kg cocaine under acute or chronic regimes (see section “Materials and Methods”). D_1_R-σ_1_R or D_1_R-σ_2_R heteromer complexes were detected by PLA using of specific antibodies against D_1_R (1/100), σ_1_R (1/100, Santa Cruz Biotechnology, Dallas, TX, United States) or σ_2_R (1/100). Confocal microscopy images (four superimposed sections) were obtained where nuclei were stained with Hoechst (1/100). Scale bar 5 μm **(A)**. Quantification of the PLA provides in the Y-axis the ratio *r* (number of red spots/cell containing spots) and, above each bar, the percentage of positive cells versus the total number of cells (blue nucleus) **(B)**. Data are the mean ± SEM of six different fields in five independent preparations. One way ANOVA and Dunnett’s multiple comparison *post hoc* test showed statistically significant differences versus control (^∗^*p* < 0.05, ^∗∗^*p* < 0.01, ^∗∗∗^*p* < 0.001), significant differences in D_1_R-σ_1_R complex amount between acute and chronic treatments (^&&&^*p* < 0.001) and significant differences in D_1_R-σ_2_R heteromer complex amount between acute and chronic treatments (^$$^*p* < 0.01). cAMP determination experiments were developed in primary cultures of striatal neurons, control **(C)**, transfected with 3 μg siRNA for σ_1_R **(D)** or 3 μg siRNA for σ_2_R **(E)**. Cultures were divided into 9 groups and pretreated with vehicle or 30 μM cocaine for different time periods (from 0.5 h to 7 days) prior to receptor activation using 200 nM SKF-81297. Basal [cAMP] is considered 100%. Values are the mean ± SEM of five different experiments. One way ANOVA followed by a Dunnett’s multiple comparison *post hoc* test showed a significant effect of treatments versus basal (^∗^*p* < 0.05, ^∗∗∗^*p* < 0.001) and a significant effect of cocaine treatments (white bars) versus cocaine non-exposed neurons (black bar) (^#^*p* < 0.05, ^##^*p* < 0.01, and ^###^*p* < 0.001).

*In situ* PLAs were performed to identify D_1_R-σ_1_R and D_1_R-σ_2_R heteroreceptor complexes in striatal sections from Sprague–Dawley rats receiving cocaine under acute or chronic regimes (see section “Materials and Methods”) (**Figure 6A**). When striatal sections of vehicle-treated animals were analyzed, it was observed that 38.5% of cells showed D_1_R-σ_1_R complexes with 2.5 red spots/cell containing spots, while only 25% of cells showed D_1_R-σ_2_R complexes with 2.1 dots/cell (**Figure 6B**). When Sprague–Dawley rats were acutely treated with cocaine, it was observed that both D_1_R-σ_1_R and D_1_R-σ_2_R complex expression increased. However, the D_1_R-σ_1_R complexes doubled its expression while D_1_R-σ_2_R complex expression suffered a slight increase (respectively, 54% of cells showed red spots with 4.5 spots/cell and 33% with 2.3 spots/cell). Interestingly, in the case of rats chronically treated with cocaine, the D_1_R-σ_1_R heteromer complex expression was not affected (34% of cells showed red spots with 2.4 spots/cell containing spots) compared to control animals, while the D_1_R-σ_2_R heteromer expression significantly increased (35% of cells containing spots with 3.4 spots/cell containing spots) (**Figure 6B**). These results indicate that acute cocaine treatment strongly increases D_1_R-σ_1_R complexes formation in striatal rat sections but chronic cocaine treatment only drives D_1_R-σ_2_R complex expression. Then, we questioned if the cocaine-induced alterations in D_1_R-σ_1_R and D_1_R-σ_2_R complex expression had signaling consequences. To do so, we analyzed SKF-81297-induced cAMP production in primary cultures of striatal neurons pretreated with vehicle or cocaine for different times (from 0.5 h to 7 days). Interestingly, we observed that at short times SKF-81297-induced cAMP levels were further increased. In agreement with results in HEK-293 cells, cocaine binding to the σ_1_R induced a positive modulation over dopamine D_1_R-mediated signaling. When primary cultures of neurons were longer exposed to cocaine (1–7 days), SKF-81297-induced increase in cAMP levels was inhibited (**Figure 6C**). Taking into account the results in HEK-293 cells such effect seems associated to D_1_R-σ_2_R complex formation and to the ability of σ_2_R to counteract the SKF-81297-induced increases of cAMP. To check whether these interpretations were correct, i.e., if sigma receptors were responsible of cocaine-induced modulations over D_1_R-mediated signaling, primary striatal neurons were transfected with siRNA specific for σ_1_R or σ_2_R. On the one hand, cocaine pretreatment (0.5 h to 7 days) blocked SKF-81297-induced accumulation of cAMP levels in primary cultures of neurons transfected with siRNA for σ_1_R, i.e., expressing D_1_R and σ_2_R (**Figure 6D**). On the other hand, 0.5 h and 2 h pretreatment of cocaine potentiated the SKF-81297-induced increases in cAMP levels in primary neurons transfected with siRNA for σ_2_R, i.e., expressing D_1_R and σ_1_R. However, longer periods of cocaine exposure (1–7 days) produced no effect (**Figure 6E**). These results suggest that in acute cocaine treatment D_1_R form heteromers mainly with σ_1_R, prevailing the D_1_R-σ_1_R-mediated signaling. In contrast, in the chronic situation, the increase of σ_1_R-D_1_ heteromer complex expression observed in acute conditions disappear but the increase in the D_1_R-σ_2_R complex expression is maintained, being the σ_2_R responsible of the cocaine modulation over D_1_R, hence prevailing the D_1_R-σ_2_R-mediated signaling.

## Discussion

Sigma receptors are relevant in cocaine addiction, because binding of cocaine to these receptors modulates dopaminergic transmission. Although cocaine can bind to both σ_1_R and σ_2_R, they are not closely related and no common structural properties have been identified. With respect to the modulation of receptor-mediated signaling, a relevant difference is revealed by the formation of heteroreceptor complexes. On the one hand, σ_1_R interacts with D_1_ and D_2_ dopamine receptors (Navarro et al., 2010; Moreno et al., 2014). Interestingly, we here report that σ_2_R may form heteromeric complexes with D_1_R but not with D_2_R.

Information on PGRMC1/σ_2_R expression in brain is partial. Intlekofer and Petersen (2011) confirmed data by Krebs et al. (2000) showing enrichment of the receptor in nuclei of the hypothalamus that are important for female reproduction. Petersen et al. (2013) in 2013, reviewed neuroanatomical data on the expression PGRMC1 and related proteins in CNS neuroendocrine nuclei. To our knowledge, information on expression in other neural regions is either absent or preliminary. Interestingly, a recently developed fluorescent probe tested in rat brain indicates that the receptor is more present in neurons than in glial cells (Zeng et al., 2016). Despite good *in vitro* properties, some of the radiolabeled probes that were developed for *in vivo* σ2 receptor imaging have not reached the final objective (Abate et al., 2013; Selivanova et al., 2015). In contrast, recently reported ^18^F-labeled PET probes, with enhanced brain uptake and σ_2_R selectivity (in mice), show promise for *in vivo* imaging of the receptor in the human brain (Wang et al., 2017). Surely these novel tools will be instrumental to achieve a more detailed mapping of the receptor in the CNS, specially in those areas in which dopamine receptors are expressed. There is, however, strong evidence of expression in the striatum; for instance, a recent report shows that receptor agonist regulate dopaminergic input into the striatum and the receptor is presynaptically expressed the *nucleus accumbens* (Klawonn et al., 2017). Furthermore, pioneering studies by Werling and colleagues showed σ_2_R involvement in control of dopamine transporter activity in striatum (Derbez et al., 2002) and that the striatal receptor was a target of cocaine (Nuwayhid and Werling, 2006).

The results presented here and those already reported (Navarro et al., 2010, 2013) show that in equivalent experimental configurations, cocaine binding to σ_1_R improves the accumulation of cAMP mediated by D_1_R and inhibits MAP kinase signaling. Cocaine, via σ_2_R, blocks D_1_-mediated cAMP accumulation and enhances MAP kinase activation. Importantly, similar results were obtained in HEK-293T cells and primary neuronal cultures.

D_1_R can form complexes and high- order heteromers by interacting simultaneously with the σ_1_ and σ_2_ receptors, σ_1_R being able to displace σ_2_R, but not vice versa. Navarro et al. (2010) reported an increase in the plasma membrane expression of σ_1_R after acute exposure to cocaine. When increase in σ_1_R levels in the plasma membrane occurs, σ_2_R is displaced from the D_1_R-σ_2_R or D_1_R-σ_2_R-σ_1_R heteroreceptor complexes. Such phenomenon results in increasing the amount of D_1_-σ_1_ heteroreceptors and D_1_R signaling whereas, as reported by Navarro et al. (2013), reducing D_2_R-mediated actions. However, in a longer exposure to cocaine, the signaling mediated by the dopamine D_1_R fits more with that occurring via a D_1_R-σ_2_R functional unit. These data suggest that the initial cocaine-induced overexpression in the plasma membrane of the σ_1_R is transient; once these levels decrease, due to internalization or other still unknown mechanisms, σ_2_ is the predominant receptor forming heteromers with dopamine D_1_R.

Motor control in the basal ganglia is achieved through a complex circuit composed of GABAergic neurons that contain mainly D_1_R (direct pathway) and GABAergic neurons containing mostly D_2_R (indirect pathway) (Grillner and Robertson, 2016). Fine motor control is achieved by a balance of dopaminergic signals, one via D_1_ receptors, which are G_s_ coupled, and another via D_2_ receptors, which are G_i_ coupled (Jenner, 1995; Gerfen, 2000). The deterioration of motor control by cocaine depends on the imbalance of the direct/indirect pathway, but the underlying mechanism remains unclear. Although the scenario is complex, cocaine is known to increase cAMP levels in cells expressing D_1_R-σ_1_R (Navarro et al., 2010). Therefore, cocaine seems to be increasing in the direct pathway the cAMP-dependent dopaminergic output, namely activation of protein kinase A and cAMP-regulated DARPP-32 phosphoprotein (Svenningsson et al., 2004). Through the same receptor (σ_1_R), cocaine leads to a deterioration of the dopaminergic performance of the indirect route (Navarro et al., 2013). In addition to the imbalance resulting from these σ_1_R-dependent effects, our results demonstrate that trimers of D_1_, σ_1,_ and σ_2_ receptors may be formed and that cocaine acting on these heteromers reduces the negative modulation exerted by the D_1_R-σ_1_R complexes.

The results here presented also show that the MAP kinase signaling pathway is particularly affected by the action of cocaine upon dopamine-sigma heteroreceptors. While in cells expressing the D_1_R-σ_1_R heteromer, cocaine decreased ERK1/2 phosphorylation, cocaine did the opposite in cells expressing the D_1_R-σ_2_R heteromer. It is known that ERKs are involved in the plastic changes induced by the consumption of drugs of abuse (Radwanska et al., 2005). In addition, the inhibition of ERK phosphorylation alters learned place-preference in a paradigm of drug-of-abuse consumption, whereas activation of ERK1/2 is necessary to establish the association between place preference and drug consumption (Valjent et al., 2006; Du et al., 2017). In this context, knocking down ERK1 has shown that enhanced ERK2 signaling and repeated exposure to the drug facilitate the plastic changes leading to drug addiction (Ferguson et al., 2006). It should be noted that the temporal pattern of MAP kinase activation in the mouse brain is differently induced by addictive or non-addictive drugs (Valjent et al., 2004). Interestingly, Zhang et al. (2017) have described that D_1_ receptor antagonists alter in cocaine-treated mice the length of *nucleus accumbens* postsynaptic densities, i.e., cocaine-induced long-term plasticity; however, the mechanism underlying this phenomenon has not been described. According to the previous reports and to our results, it may be suggested that potentiation of MAP kinase pathway mediated by the D_1_R-σ_2_R heteromer may be the mechanism by which the σ_2_R would induce long term neuronal plasticity. The predominant role of the σ_1_R in acute cocaine use shifts to a more relevant role of σ_2_R in the chronic condition leading to the establishment of addiction. In any case, the relative expression of the two receptors in a given neuron seems important in determining the fate of the cell when the drug of abuse is consumed.

In acute cocaine exposure, σ_1_R modulation of D_1_R-mediated signaling prevails, but in longer exposures, there is a shift to regulation by σ_2_R. Recently, Singer et al. (2017) have determined that neuronal plasticity initiate 2 h after cocaine exposure. The mechanism of action described in this paper cannot explain some of the results reported by Matsumoto et al. (2007) and Lever et al. (2014) who report that σ_2_R receptor antagonists block the effects of cocaine-induced hyperlocomotion. It should be, however, noted that a recent report show benefits of a σ_2_R -selective agonist, siramesine, for decreasing cocaine effects via reduction of dopaminergic and glutamatergic input to the striatum (Klawonn et al., 2017). On the one hand, it is a reasonable assumption that σ_1_R is more involved in the regulation of D_1_R signaling at acute exposure. However, it remains to be determined whether some of the results reported on the impact of cocaine on locomotion are due to the use of non-selective ligands, that is, ligands that can bind to both sigma receptors and alter their function. Alternatively, it may happen that σ_2_R is also affecting the direct route in acute conditions. What our results undoubtedly indicate is that σ_2_R becomes the main player in conditions of chronic exposure to the drug. In summary, some of the addictive and motor actions of cocaine are the result of a balance between cocaine-σ_1_R versus cocaine-σ_2_R impact on activation of D_1_R and D_2_R (and D_1_R-D_2_R, see Perreault et al., 2016) in *ad hoc* CNS circuits.

## Materials and Methods

### Reagents

Cocaine-chlorhydrate was provided by the Spanish *Agencia del Medicamento* (Ref. n°: 2003C00220). σ_2_R agonist, 1-Cyclohexyl-4-[3-(1,2,3,4-tetrahydro-5-methoxy-1-naphthalenyl)propyl]piperazine dihydrochloride (PB-28), D_1_R agonist (±)-6-Chloro-2,3,4,5-tetrahydro-1-phenyl-1H-3-benzazepine hydrobromide (SKF-81297) and D_2_R agonist, sumanirole, were purchased from Tocris, Bristol, United Kingdom.

### Fusion Proteins and Expression Vectors

cDNAs for human versions of D_1_R, D_2_R, σ_1_R, or σ_2_R cloned into pcDNA3.1, were amplified without their stop codons using sense and antisense primers harboring: *EcoRI* and *KpnI* sites to subclone D_1_R, D_2_R, σ_1_R, and σ_2_R in pcDNA3.1Rluc vector (p*Rluc*-N1, PerkinElmer Life and Analytical Sciences, Wellesley, MA, United States) or *HindIII* and *BamHI* sites to clone D_1_R, D_2_R, σ_1_R, and σ_2_R in pEYFP-N1 vector (enhanced yellow variant of GFP; Clontech), or *EcoRI* and *BamHI* sites to clone σ_2_R in a cherry-containing vector (pcDNA3.1Cherry). Amplified fragments were subcloned to be in-frame with restriction sites for p*Rluc*-N1, pEYFP-N1, or pcDNA3.1Cherry vectors to provide plasmids that express proteins fused to *Renilla* Luciferase (D_1_R-Rluc, D_2_R-Rluc, σ_1_R-Rluc, and σ_2_R-Rluc), YFP (D_1_R-YFP, D_2_R-YFP, σ_1_R-YFP, and σ_2_R-YFP) or cherry (σ_2_R-Cherry) at the C-terminal end.

### Cell Lines and Transient Transfection

HEK-293T human embryonic kidney cells were grown at 37°C in a humid atmosphere with 5% CO_2_ in Dulbecco’s modified Eagle’s medium (DMEM) (Gibco, Thermo Fischer Scientific, Madrid, Spain) supplemented with 2 mM L-glutamine, 100 μl/ml sodium pyruvate, 100 U/ml penicillin/streptomycin, MEM Non-Essential Amino Acid Solution (1/100) and 5% (v/v) heat inactivated foetal bovine serum (FBS) (all supplements were from Invitrogen, Paisley, Scotland, United Kingdom). Cells were transiently transfected with constructs encoding for receptors, fusion proteins, and/or siRNAs by the polyethylenimine (PEI; Sigma–Aldrich, St. Louis, MO, United States) method. Transfected cells were incubated in serum-free medium that after 4 h was replaced by complete medium. Experiments were carried out 48 h later.

### Neuronal Primary Cultures

Primary cultures of striatal neurons were obtained from 19-day embryos of Sprague–Dawley rats. Cells were isolated as described in Hradsky et al. (2013) and plated at a confluence of 40,000 cells/0.32 cm^2^. Cells were maintained for 12 days in Neurobasal medium supplemented with 2 mM L-glutamine, 100 U/ml penicillin/streptomycin, and 2% (v/v) B27 supplement (Gibco) in 6-well plates. When indicated, cells were transiently transfected with the corresponding siRNA (3 μg plasmid siRNA per well) using the Lipofectamine^TM^ 2000 (Invitrogen, Life Technologies, Darmstadt, Germany). Transfected cells were incubated in serum-free medium that after 4 h was replaced by complete medium. Experiments were carried out 48 h later.

### Cocaine Treatment of Sprague–Dawley Rats

Male Sprague–Dawley rats weighing 200–220 g were selected for the experiments. Rats were kept in controlled environment with 12 h light-dark cycle at 21°C room temperature. Food and water were provided *ad libitum*. Experimental procedures were approved by the Bioethical Committee of the Faculty of Biological Sciences of the Pontificia Universidad Católica de Chile and follow the international guidelines (NIH Guide for the Care and Use of Laboratory Animals). Rats were housed and handled in colony for three days, and then were divided in two experimental groups: acute and chronic, with respective saline controls. Chronic cocaine administration consisted in two injections of cocaine (15 mg/kg, i.p.) per day for 14 days at 11:00 A.M. and 5:00 P.M., as described by Liu et al. (2005). Acute cocaine administration consisted of two injections of cocaine (15 mg/kg, i.p.) for only one day. The same protocol of administration was used in control animals receiving saline injections. Rats were sacrificed 17 h. after the last saline or cocaine injection following the protocol of Liu et al. (2005). Cocaine HCl was donated by the National Institute on Drug Abuse (NIDA, United States).

### Immunocytochemistry

HEK-293T cells were treated with 30 μM cocaine or vehicle for 30 min, then were washed with PBS, fixed in 4% paraformaldehyde for 15 min and washed with PBS containing 20 mM glycine to quench free aldehyde groups. After permeabilization with PBS-glycine buffer containing 0.2% Triton X-100 for 5 min, cells were blocked with PBS containing 1% bovine serum albumin (BSA) for 1 h at room temperature. D_1_R-YFP and D_2_R-YFP were detected by its own fluorescence (wavelength 530 nm), and σ_2_R-Rluc was stained using a primary anti-Rluc mouse monoclonal antibody (1/200, Millipore, CA, United States) for 1 h, washed and stained for another hour with the secondary Cy3-conjugated donkey anti-mouse antibody (1/200, Jackson Immunoresearch Laboratories, West Grove, PA, United States). Nuclei were stained with Hoechst (1/100, Sigma–Aldrich, St. Louis, MO, United States) and then samples were rinsed several times and mounted with Mowiol 30% (Calbiochem). Images were taken using a Leica SP2 confocal microscope (Leica Microsystems, Mannheim, Germany).

### Proximity Ligation Assay

For PLAs, HEK-293T cells or primary cultures of striatal neurons were grown on glass coverslips while cocaine-administered rat sections were mounted and dried directly on glass coverslips. Samples were treated with 30 μM cocaine or vehicle for 30 min, then were washed with PBS and fixed in 4% paraformaldehyde for 15 min, washed with PBS containing 20 mM glycine, permeabilized with the same buffer containing 0.05% Triton X-100 for 5 min, and washed with PBS. Then, samples were incubated at 37°C with the blocking solution for 1 h. Heteromers were detected using the Duolink *in situ* PLA detection kit (OLink Bioscience, Bioscience, Uppsala, Sweden) following the instructions of the supplier. To detect D_1_R-σ_2_R or D_2_R-σ_2_R heteromers, cells and primary cultures were incubated overnight with anti D_1_R (1/100), anti σ_2_R (1/100) and Hoechst (1/100), or anti D_2_R (1/100), anti σ_2_R and Hoechst. Samples were processed using PLA probes that bind to the primary antibodies (Duolink II PLA probe anti-mouse plus and Duolink II PLA probe anti-goat minus). Images were taken using a Leica SP2 confocal microscope (Leica Microsystems, Mannheim, Germany) equipped with an apochromatic 63× oil-immersion objective (numerical aperture 1.4) and 405 and 561 nm laser lines. For each field of view a stack of two channels (one per staining) and 4–6 Z stacks with a step size of 1 μm were acquired. Quantification of the number of cells containing one or more red spots versus total cells (blue nuclei) and, in cells containing spots, of the number of red spots/cell ratio, was conducted using dedicated software known as Duolink ImageTool (ref: DUO90806, Sigma-Olink). This software has been developed for quantification of PLA signals and cell nuclei in images generated from fluorescence microscopy. One-way ANOVA followed by Dunnett’s *post hoc* multiple comparison test was used for statistical analysis.

### Resonance Energy Transfer

For bioluminescence resonance energy transfer (BRET), HEK-293T cells were transiently cotransfected with a constant amount of cDNA encoding for proteins fused to Rluc and increasing amounts of cDNAs corresponding to proteins fused to YFP (see figure legends). To normalize the number of cells, protein concentration was determined using a Bradford assay kit (Bio-Rad, Munich, Germany) using BSA dilutions as standards. To quantify protein YFP expression, cells (20 μg of protein) were distributed in 96-well plates (black plates with a transparent bottom), and fluorescence was read in the FluoStar Optima Fluorimeter (BMG Labtech, Offenburg, Germany) equipped with a high-energy xenon flash lamp, using a 10-nm bandwidth excitation filter at 400 nm reading. Protein fluorescence expression was determined as fluorescence of the sample minus the fluorescence of cells expressing the BRET donor alone. For BRET measurements, the equivalent of 20 μg of cell suspension was distributed in 96-well plates (Corning 3600, white plates; Sigma), and coelenterazine H (5 μM; Invitrogen) was added. After 1 min, readings were obtained using a Mithras LB 940 (Berthold Technologies), which allows the integration of the signals detected in the short-wavelength filter at 485 nm and the long-wavelength filter at 530 nm. To quantify protein-Rluc luminescence, readings were also performed 10 min after addition of coelenterazine H. For SRET assays, cells were transiently cotransfected with constant amounts of cDNA encoding for both receptor fused to Rluc and YFP proteins, and with increasing amounts of cDNA corresponding to the receptor fused to cherry protein. After 48 h of transfection, quantification was performed in parallel in aliquots of transfected cells (20 μg of protein): quantification of receptor YFP or receptor Rluc expression was performed as indicated for BRET experiments. Quantification of receptor-Cherry expression, cells were distributed in 96-well plates (Corning black plates with a transparent bottom), and fluorescence was read in the FluoStar Optima Fluorimeter using a 10 nm bandwidth excitation filter at 590 nm reading. For SRET quantification, cells were distributed in 96-well plates (black plates with transparent bottom), and coelenterazine H (5 μM) was added. After 1 min, the readings were collected using a FluoStar Optima Fluorimeter, which allows the integration of the signals detected in the short-wavelength filter at 530 nm and the long-wavelength filter at 590 nm. Net BRET and net SRET were defined as [(long-wavelength emission)/(short-wavelength emission)] – C_f_, where C_f_ corresponds to [(long-wavelength emission)/(short-wavelength emission)] for the Rluc construct expressed alone in the same experiment. Both fluorescence and luminescence were measured before every experiment to confirm similar donor expressions (∼100,000 bioluminescence units) while monitoring the increase in acceptor expression (1,000–40,000 fluorescence units). BRET or SRET was expressed as, respectively, milliBRET (mBU) or milliSRET (mSU) units (net BRET or SRET × 1,000). Data were fitted to a nonlinear regression equation, assuming a single-phase saturation curve with GraphPad Prism software (GraphPad Software). The relative amount of BRET or SRET is given as a function of 100× the ratio between the fluorescence of the acceptor (YFP or cherry) and the luciferase activity of the donor (Rluc).

### cAMP Determination

cAMP levels were assayed with different forskolin concentrations and cell densities to select the most appropriate conditions of the assay, which were 0.5 μM forskolin and 5,000 HEK-293T cells or 7,500 neurons. Transfected HEK-293T cells or neurons were incubated in serum-free medium for 3 h before the experiment. Then, cells were placed in 384-well microplates in medium containing 50 μM zardaverine (Tocris Bioscience). Cells were then preincubated with vehicle, the σ_2_R agonist, PB-28 (300 nM) or cocaine (30 μM) for 15 min, followed by dopaminergic stimulation with the D_1_R agonist, SKF-81297 (200 nM), the D_2_R agonist, sumanirole (500 nM) or vehicle. After another incubation period of 15 min, 0.5 μM forskolin or vehicle were added. Readings were performed 15 min later by the use of a homogeneous time-resolved fluorescence energy transfer (HTRF) method requiring the Lance Ultra cAMP kit (PerkinElmer) and fluorescence readings (at 635 nm) in a PHERAstar Flagship microplate equipped with a time-resolved fluorescence optical module (BMG Labtech).

### ERK1/2 Phosphorylation

To determine ERK1/2 phosphorylation, 40,000 HEK-293T cells/well or 50,000 neurons/well were plated in transparent Deltalab 96-well plates and kept in the incubator for 48 h. The medium was substituted by serum-free DMEM medium for 2–4 h before initiating the experiment. Then, HEK-293T cells and striatal neurons were pretreated for 10 min at 25°C with vehicle, PB-28 (300 nM) or cocaine (30 μM) followed by the addition of 200 nM SKF-81297, the D_1_R specific agonist. 10 min after activation, cells/neurons were placed on ice and washed twice with cold PBS before the addition of 30 μl of lysis buffer for 15 min. Supernatants (10 μl) were placed in white ProxiPlate 384-well microplates, and ERK1/2 phosphorylation was determined using the AlphaScreen^®^SureFire^®^ kit (Perkin Elmer) and the EnSpire^®^ Multimode Plate Reader (PerkinElmer, Waltham, MA, United States).

### Label-Free Dynamic Mass Redistribution Assays (DMR)

HEK-293T cells and neuronal primary cultures were seeded in 384-well sensor microplates for 24 h before the assay to obtain 70–80% confluent monolayers constituted by 5,000 HEK-293T cells or 14,000 neurons per well. Previous to the assay, cells were washed twice with assay buffer (HBSS with 20 mM HEPES and 0.1% DMSO, pH 7.15) and incubated for 2 h in 40 μl/well of assay-buffer in the reader at 24°C. Hereafter, the sensor plate was scanned and a baseline optical signature was recorded before adding (10 μl) vehicle, cocaine (30 μM) or PB-28 (300 nM) for 30 min followed by SKF-81297 (200 nM) addition. All compounds dissolved in assay buffer. Then, DMR responses were monitored for at least 3,600 s using an EnSpire^®^ Multimode Plate Reader (PerkinElmer Life and Analytical Sciences, Waltham, MA, United States). Sensitive measurements of changes in local optical density mimicking cellular mass movements induced upon receptor activation were detected using EnSpire Workstation Software v4.10, and curves were normalized with respect to the baseline.

### Data Analysis

The data in graphs are the mean ± SEM. The test of Kolmogorov–Smirnov with the correction of Lilliefors was used to evaluate normal distribution and the test of Levene to evaluate the homogeneity of variance. Parametric statistic methods were used, because results in the different groups showed normality and homogeneity of variance. Significance was analyzed by one-way ANOVA, followed by Dunnett’s multiple comparison *post hoc* test. GraphPad Prism software version 5 was used for the statistical analysis. Significant differences were considered when *p* < 0.05.

## Author Contributions

GN, EC, and RF designed the experiments and directed the project. DA did many of the cell and molecular assays and did the statistics of the results in the laboratory of the University of Barcelona. KG designed the experiments to obtain cocaine-treated animals in the laboratory in Chile. MM and IV-Q administered drugs to animals, prepared the brain sections, and performed the immunological-based histochemical assays. GN and RF wrote the first draft of the manuscript, which was further edited by DA, KG, EC, and IV-Q.

## Conflict of Interest Statement

The authors declare that the research was conducted in the absence of any commercial or financial relationships that could be construed as a potential conflict of interest.
